# Unveiling the Uncommon: Rickettsial Infections Promoting Sudden-Onset Parkinsonism

**DOI:** 10.7759/cureus.80570

**Published:** 2025-03-14

**Authors:** Fatemeh Jafari Roshan-Zamir, Josna A Gigi, Iva Conde, Patrick Hayes

**Affiliations:** 1 Neurology, William Carey University College of Osteopathic Medicine, Hattiesburg, USA; 2 Internal Medicine, William Carey University College of Osteopathic Medicine, Hattiesburg, USA; 3 Psychiatry, Elite Medical Wellness, Lake Charles, USA

**Keywords:** drug-induced parkinsonism, movement disorder, parkinsonism, rickettsia rickettsii, rocky mountain spotted fever

## Abstract

It is known that various infections can lead to neuroinflammation and parkinsonism. Parkinsonism encompasses a range of neurodegenerative disorders that can present with symptoms like tremors or muscle rigidity. The following case describes a rare instance of parkinsonism that was induced by Rocky Mountain Spotted Fever (*Rickettsia rickettsii*). While parkinsonism has been found to be caused by certain viruses, it is uncommon for Rickettsial infections to be the inciting agent. This case sheds light on the evidence-based management of *Rickettsia*-induced parkinsonism (RIP) and movement disorders.

A 60-year-old male with intellectual and developmental disabilities (IDD) presented with a shuffling gait, stooped posture, bilateral hand tremors, and minimal facial movement. Antibody testing confirmed *Rickettsia*, with no other clear cause. He was treated with a seven-day course of 100 mg doxycycline, plus a six-month course of 100 mg amantadine and 0.5 mg clonazepam, both twice daily. At follow-up, he showed marked improvement, with a normal gait, upright posture, clearer speech, and no tremors. This case exemplifies the intricacies associated with Rickettsial infections. However, upon accurate diagnosis, appropriate treatment can be effectively administered.

Our patient experienced sudden-onset parkinsonism following a recent Rickettsial infection. Similar cases that have been documented in the scientific literature have reported the *Orientia* species as the infective pathogen. Additionally, these reported cases were predominantly observed in Asia, while our patient resides in the United States. This case can guide clinicians treating movement difficulties after Rickettsial infections and raise awareness of the existence of the rare etiology, treatment, and prognosis.

## Introduction

The *Rickettsia* genus comprises obligate intracellular pathogens that depend on host cell replication for survival. These bacteria are transmitted to humans through arthropod vectors such as ticks, fleas, and lice, infiltrating multiple organ systems and leading to various illnesses. *Rickettsia* species demonstrate a strong affinity for the central nervous system (CNS), contributing to neurological complications such as meningitis, encephalitis, and, in rare cases, *Rickettsia*-induced parkinsonism (RIP). Mechanistically, Rickettsial infections can promote neuronal degeneration through adenosine triphosphate (ATP) depletion, neuroinflammation, and endothelial dysfunction, ultimately affecting basal ganglia circuits involved in motor control [1].

Rickettsial pathogens are classified into three major groups: the typhus group, the spotted fever group, and the scrub typhus group [2]. While *Orientia tsutsugamushi* (scrub typhus) has been documented as a potential cause of parkinsonian features in Asia, such as in a Sri Lankan case where a 62-year-old male exhibited reversible parkinsonism following *O. tsutsugamushi* infection, the association between *Rickettsia rickettsii* (Rocky Mountain Spotted Fever, or RMSF) and movement disorders remains underexplored in the United States.

RMSF, primarily transmitted by ticks, is a widespread disease beyond the Rocky Mountain region, particularly in the U.S. Upon entry into vascular endothelial cells, *R. rickettsii* replicates within blood vessels, triggering endothelial proliferation, neuroinflammation, and immune activation, which may disrupt dopaminergic pathways and contribute to parkinsonian symptoms [3]. However, diagnosing RMSF can be challenging due to the absence of characteristic eschars and the nonspecificity of serological findings, leading to potential underdiagnosis in non-endemic regions.

Although Rickettsial infections can induce Parkinson-like symptoms, they must be differentiated from other reversible and neurodegenerative causes of parkinsonism. Drug-induced parkinsonism, for example, results from dopamine receptor blockade by medications such as antipsychotics and calcium-channel blockers, leading to tremors, rigidity, and bradykinesia [4]. This distinction is crucial in evaluating movement disorders associated with infectious etiologies.

This case report describes a 60-year-old male with intellectual and developmental disabilities (IDD) who developed presumed RMSF-induced parkinsonism. The case underscores the need for heightened clinical suspicion in atypical presentations and non-endemic regions. By examining this case, we aim to contribute to the growing understanding of infection-associated movement disorders and highlight diagnostic challenges in recognizing RMSF-induced neurological manifestations.

## Case presentation

A 60-year-old male reported a past medical history significant for moderate IDD, hypertension, and bipolar and seizure disorders. A long-term patient under care for medication management, he was described as “nice and compliant” by staff at his residential care facility, prior to symptom onset. 

At a quarterly follow-up, the patient reported experiencing several falls, balance issues, an inability to bend his knees, and an overall shuffling gait. The patient’s caregiver reported him feeling as though he had to “drag his feet” when walking, and the symptoms had a sudden onset. Notably, the patient had a brief period of flu-like symptoms almost three weeks prior, which resolved after two to three days, according to his caregiver. 

On physical examination, the patient exhibited minimal facial movements, a resting tremor in his hands that showed parkinsonian frequency and characteristics, an increased shuffle in his gait, a forward-tilted trunk, and a stooping posture. These findings suggested the development of acute Parkinson-like features, characterized by bradykinesia, rigidity, and tremor.

Given the patient's complex medical history and the emergence of these new neurological symptoms, further evaluation and management were warranted. Differential diagnoses included infection/medication-induced parkinsonism, Parkinson's disease, or other neurodegenerative disorders. Close monitoring, multidisciplinary collaboration, and targeted interventions were essential in providing comprehensive care for this patient. Due to the abrupt onset of symptoms and change from baseline, the patient was referred to a neurologist who ordered Rickettsial titers.

Upon testing positive for Rickettsial titers (Table 1), the patient’s history was further evaluated to eliminate other possible etiologies. Table 2 presents the patient's results following a more in-depth examination. These results indicate normal inflammatory markers, ruling out concern for a viral source. Additionally, they show that his vitamin levels and thyroid panel are within the normal range. After a detailed literature review, it was concluded that Rickettsial infections can be diagnosed based on serological response and a high degree of clinical suspicion, and that Rickettsial infections can precipitate Parkinson-like features. Additional imaging was not performed, as it was not warranted according to prior literature. Informed consent was appropriately obtained from the patient, including permission for the publication of case details.

**Table 1:** Rickettsial infection antibodies. Rocky Mountain Spotted Fever and Scrub Typhus were tested. The Spotted Fever test came back positive in this patient.

Test	Result	Normal Value
*Rickettsia* Spotted Group IgG	1:256 (High)	<1:64
*Rickettsia* Spotted Group IgM	<1:64	<1:64
*Rickettsia* Typhus Group IgG	<1:64	<1:64
*Rickettsia* Typhus Group IgM	<1:64	<1:64

**Table 2:** Detailed lab-work to consider other etiologies. Other etiologies that could be attributed to encephalopathy were investigated, and these were used to rule out the source of infection.

Test	Result	Normal Value
Rheumatoid Factor	<10 IU/mL	<14
C-Reactive Protein	<0.3 mg/dL	<0.5
Sedimentation Rate	2 mm/hour	0-15
Lyme Total Antibody Reflex IgG/IgM	0.24	<0.90
West Nile Virus IgM	0.09	<=0.89
West Nile Virus IgG	0.45	<=1.29
West Equine Encephalitis IgG	<1:16	<1:16
West Equine Encephalitis IgM	<1:16	<1:16
East Equine Encephalitis IgG	<1:16	<1:16
East Equine Encephalitis IgM	<1:16	<1:16
California Encephalitis IgG	<1:16	<1:16
California Encephalitis IgM	<1:16	<1:16
St. Louis Encephalitis IgG	<1:16	<1:16
St. Louis Encephalitis IgM	<1:16	<1:16
Rapid Plasma Reagin (RPR)	Non-reactive	Non-reactive
Vitamin D, 25 OH	20 ng/mL (Suboptimal)	30-100
Vitamin C	23 umol/L	23-114
Vitamin B12	702 pg/mL	200-950
Vitamin B6	90 nmol/L	20-125
Vitamin B2	8 nmol/L	5-50
Vitamin B1	75 nmol/L	64-201
Folic Acid	9.6 ug/L	>=6.0
Thyroid-Stimulating Hormone (TSH), Third Generation	1.170 UIU/mL	0.400-4.100
Free T3	2.8 pg/mL	2.2-4.2
Free T4	0.99 ng/dL	0.80-1.90
Thyroid Peroxidase Antibody	10 IU/mL	<=34
Urinalysis		
Color	Yellow	Yellow/Straw
Appearance	Clear	Clear
Specific Gravity	1.020	1.005-1.035
Leukocyte Esterase	Negative	Negative
Nitrite	Negative	Negative
pH	6.5	5.0-9.0
Protein	Negative	Negative
Glucose	Negative	Negative
Ketones	Negative	Negative
Urobilogen	1.0 mg/dL	<=2.0
Bilirubin	Negative	Negative
Occult Blood	Negative	Negative

The patient was prescribed doxycycline 100 mg orally every 12 hours for seven days to address the presumed Rickettsial infection. To manage the acute Parkinson-like features, the patient was prescribed amantadine 100 mg orally twice daily and clonazepam 0.5 mg orally twice daily.

At the six-week follow-up appointment, significant improvements were observed in the patient's clinical presentation. His gait had notably improved, with the patient now walking fully upright. Speech clarity had also improved, and there were minimal observable tremors. These positive changes indicated a favorable response to the pharmacological intervention. Two weeks following the initial improvements, both the patient and his caregiver reported the complete disappearance of tremors. The patient's speech had returned to baseline, and his gait remained stable and sustained. These outcomes indicated the resolution of the acute parkinsonism symptoms.

Given the positive response to treatment and sustained improvement in clinical status, the decision was made to continue the administration of amantadine and clonazepam for a total duration of six months. Follow-ups were scheduled more frequently to monitor the re-emergence of symptoms. Since the completion of these medications, the patient has not exhibited any Parkinson-like features. Amantadine and clonazepam were discontinued after six months, as the Parkinson-like features were no longer present. This highlights that the patient's symptoms were most likely caused by a Rickettsial infection presenting with Parkinson-like characteristics, and that, with successful treatment, the need for medication has been eliminated.

Overall, the management involving antibiotic therapy for the underlying infection, along with targeted treatment for the neurological symptoms, led to significant clinical improvement. Close monitoring and adherence to the prescribed regimen played crucial roles in achieving successful patient outcomes.

## Discussion

*Rickettsia* is a common cause of a host of illnesses. However, due to nonspecific symptomatology, it is often underdiagnosed. Most Rickettsial infections are highly curable, especially early in the disease process [5]. Rickettsiosis can also lead to neuroinflammation, which can manifest as meningoencephalitis, ocular abnormalities, and even sensorineural hearing loss [6]. As outlined in Table 3, various species of *Rickettsia* can exhibit a broad spectrum of neurological presentations [3].

**Table 3:** Neurological features of Rickettsial species. Table credit: [3] Clinical manifestations of various strains of *Rickettsia* are described in this table. As seen here, neuroinflammation is a common complication of this species.

Group	Organism	Neurological Features
Spotted Fever Group		
Rocky Mountain Spotted Fever	Rickettsia rickettsii	Encephalitis, meningitis, meningoencephalitis, deafness, central nerve palsies, Guillain-Barré polyneuropathies
Mediterranean Spotted Fever	Rickettsia conorii	Encephalitis, meningitis, meningoencephalitis, deafness, central nerve palsies, Guillain-Barré polyneuropathies
Typhus		
Epidemic Typhus	Rickettsia prowazekii	Encephalitis
Murine Typhus	Rickettsia typhi	Encephalitis, less frequent than *Rickettsia prowazekii*
Scrub Typhus	Orientia tsutsugamushi	Encephalitis, meningitis, deafness

In our case, the patient exhibited a sudden onset of posture changes, balance impairment, shuffling gait, resting hand tremors, and diminished facial expressions. The rapid worsening of symptoms led clinicians to investigate potential acute causes, prompting a comprehensive diagnostic evaluation to identify a possible source of infection. Rickettsial antibody detection was pivotal in identifying RIP secondary to a recent exposure. The question arises: when should a clinician consider testing for a tick-borne infection in the presence of parkinsonism symptoms, rather than solely focusing on neurological causes? A key consideration is the onset and progression rate of symptoms. Parkinson's disease typically manifests as a gradual, progressive disorder characterized by the degeneration of dopaminergic neurons in the midbrain [7]. As dopamine depletion progresses within the basal ganglia, patients develop clinical features over time. However, in cases of rapid symptom onset, particularly in rural populations, assessing for tick-borne infections - such as by checking for Rickettsial IgM and IgG antibody titers - is warranted. In our patient, prompt treatment with doxycycline resulted in rapid recovery. A benefit of considering RIP is the low cost and minimal risk of drawing the Rickettsial titers.

This case highlights the necessity for clinicians to remain vigilant regarding diverse etiologies when assessing patients with Parkinson-like features. A comprehensive diagnostic approach is indispensable in distinguishing idiopathic Parkinson's disease from alternative causes, facilitating appropriate management and improving patient outcomes. Collaboration among primary clinicians, neurologists, and allied healthcare professionals is essential in navigating the diagnostic complexities inherent in parkinsonian syndromes, including RIP.

## Conclusions

We presented a case of abrupt-onset Parkinson-like features that followed a presumed Rickettsial infection in a patient with a past medical history of moderate IDD, bipolar disorder, hypertension, and seizure disorder. The patient had classic features of rigidity, stooped posture, and shuffling gait that resolved with prompt diagnosis and management. Medications and conditions can precipitate or make patients more likely to exhibit Parkinson-like features. Infections caused by various organisms, like *Rickettsia* or *Orientia*, for example, can precipitate these symptoms. Additionally, drugs that block dopaminergic pathways, like antipsychotics, can also trigger Parkinson-like features. This case serves as a reminder to physicians and caregivers to be aware of Parkinson-like features and the timing of symptom onset in high-risk populations. When a patient presents with symptoms suspicious for Parkinson’s disease, keep in mind that, depending on the severity and timing of the features, multiple etiologies can cause reversible Parkinson-like features as well. Early diagnosis and treatment can prevent permanent neurological damage, so clinicians should have a high index of suspicion for this disease presentation.
